# A phase I study to evaluate the safety, tolerability, and pharmacokinetics of HEC113995PA·H_2_O, a novel dual-acting serotonergic antidepressant, in healthy subjects

**DOI:** 10.3389/fphar.2025.1500974

**Published:** 2025-03-26

**Authors:** Xue Wu, Qingqing Wu, Qichen Ding, Yulei Zhuang, Lin Luo, Yingjun Zhang, Li Deng, Chuanfei Jin, Xue Li, Zhangma Huang, Haiping Qin, Liang Xin, Qian Chen, Jingying Jia, Yanmei Liu

**Affiliations:** ^1^ Drug Clinical Trial Center, Shanghai Xuhui Central Hospital, Shanghai, China; ^2^ Shanghai Engineering Research Center of Phase I Clinical Research & Quality Consistency Evaluation for Drugs, Shanghai, China; ^3^ Shanghai Institute of Clinical Mass Spectrometry, Shanghai, China; ^4^ Sunshine Lake Pharma Co., LTD., Guangdong, China; ^5^ ED (Emergency Department) Emergency Ward, Shanghai Xuhui Central Hospital, Shanghai, China

**Keywords:** HEC113995PA, H_2_O, SPARI, safety, pharmacokinetics, food effect, healthy subjects

## Abstract

**Purpose:**

HEC113995PA·H_2_O is a novel, potent and selective serotonin (5-HT) reuptake inhibitor and a 5-HT1A receptor partial agonist, and thus is categorized as a serotonin partial agonist-reuptake inhibitor. The objective of this study was to evaluate the safety, tolerability, and pharmacokinetics of HEC113995PA·H_2_O in healthy subjects after single and multiple dosing, as well as the food effect on pharmacokinetics and safety of HEC113995PA·H_2_O.

**Methods:**

The entire study was comprised of three parts: Part I (single ascending-dose study), Part II (food effect study), and Part III (multiple ascending-dose study). A total of 121 healthy subjects were enrolled in the study. HEC113995PA·H_2_O tablet or placebo was administered per protocol requirements. Blood samples were collected at the designated time points for pharmacokinetic analysis. Safety was assessed by clinical examinations and adverse events.

**Results:**

In Part I, AUC and C_max_ were found to by and large linear within the 2.5–80 mg dose range. t_1/2_ of HEC113995PA·H_2_O was 27.17∼38.58 h. In Part II, we revealed that HEC113995PA·H_2_O administration post meal could increase C_max_ and AUC_0-t_. In Part III, multiple administration led to accumulated body exposure and the PK of healthy subjects reached a steady state after 7 days of continuous administration in each dose group.

**Conclusion:**

HEC113995PA·H_2_O was safe and generally well-tolerated in healthy subjects. Based on the pharmacokinetic and safety data mentioned above, we expect that postprandial administration will favorably increase drug concentrations in the body and reduce gastrointestinal adverse events.

## 1 Introduction

Depressive disorder (DD) is a common mental illness, with major depressive disorder (MDD) as the most prevalent subtype. DD is characterized by some common clinical symptoms including low mood, lack of pleasure, slow thinking, reduced energy, and sleep disturbances. These symptoms may be accompanied with psychotic symptoms and/or physical discomfort. Individuals with severe symptoms may experience suicidal thoughts or behaviors (Otte et al., 2016). DD is highly prevalent and morbidity continues to increase. A study by the World Health Organization (WHO) revealed an estimate of 322 million DD cases globally, accounting for 4.4% of the world’s population (Friedrich, 2017). Currently, the etiology and pathogenesis of depressive disorders remain incompletely understood. Some hypotheses suggest that the pathogenesis of depressive disorders may involve various factors, including psychosocial, genetic, neurobiochemistrical, neuroendocrine factors and brain imaging changes (Chiriţă et al., 2015; Fakhoury, 2015). A Nature study reported that DD is the most prevalent cause of years lost to disability (YLD) among major diseases, causing a significant disease burden worldwide (Smith, 2014).

Pharmaceutical therapy is the most common and fundamental strategy for combating depression. Current frontline medications used in clinical practice include Selective Serotonin Reuptake Inhibitors (SSRIs), such as escitalopram, paroxetine, sertraline, fluoxetine, and fluvoxamine, Serotonin and Norepinephrine Reuptake Inhibitors (SNRIs), including venlafaxine and duloxetine, and Norepinephrine-Dopamine Reuptake Inhibitors (NaSSA) such as mirtazapine, etc. (Sanchez et al., 2014; Bousman et al., 2023) Some recently introduced antidepressants like vortioxetine and agomelatine also showed widespread clinical use. Antidepressants elicit their effects through their actions on neurotransmitters, and because of the widespread distribution of neurotransmitter receptors throughout the human body, the adverse effects of antidepressants are commonly observed. The side effects of SSRIs typically include diarrhea, dizziness, dry mouth, headache, nausea, sexual dysfunction, sweating, tremors, and weight gain. The adverse effects of different medications may vary greatly. For instance, compared to fluvoxamine, escitalopram has a higher incidence of treatment-associate sexual dysfunction. Paroxetine is more likely to cause withdrawal symptoms and sexual dysfunction than fluvoxamine. Paroxetine is more prone to causing weight gain, in comparison to escitalopram. Escitalopram has a higher likelihood of causing diarrhea than other SSRIs (Revet et al., 2020; Yamada et al., 2023; Thase et al., 2016). Due to their actions on multiple receptors, these medications may easily result in adverse effects such as restlessness, hypertension, and headaches at high doses. Hence, there is a pressing demand for novel medicines with clear efficacy and excellent safety for clinical use.

**FIGURE 1:**
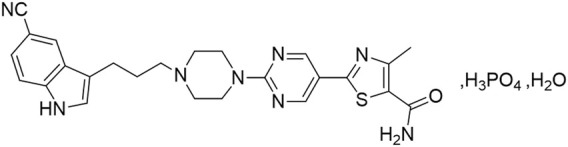
Chemical structure diagram of HEC113995PA·H_2_O.

HEC113995PA·H_2_O is a novel, orally administered, highly specific small molecule that serves as a dual-action compound, acting as both a serotonin transporter (5-HT/Serotonin Transporter, SERT) inhibitor and a partial agonist for the 5-HT1A receptors. The molecular structure of this compound is shown in Figure 1. It appears as an off-white to yellow solid with slight hygroscopicity, exhibiting acid-base ionization constants pKa1 of 6.46 and pKa2 of 7.37. The compound is readily soluble in dimethyl sulfoxide (DMSO), soluble in N,N-dimethylacetamide (DMA), and practically insoluble in dichloromethane, acetone, acetonitrile, methanol, 95% ethanol, or water. This compound belongs to the family of Serotonin partial agonist and reuptake inhibitors (SPARIs). SERT fine-tunes the serotonin neurotransmission by determining the quantity and duration of synaptic signals mediated by post-synaptic receptors through the removal of serotonin from the synaptic cleft. SERT inhibitors exert their anti-depressant effects by selectively inhibiting the activity of SERT and increasing the concentration of serotonin in the synaptic cleft. In addition, 5-HT1A receptor partial agonists exert inhibitory regulatory effects on 5-HT neurons by acting on the 5-HT1A receptors distributed on the presynaptic membrane. Through selectively partially agonizing 5-HT1A autoreceptors and inducing their rapid desensitization, 5-HT1A receptor partial agonists inhibit the activation of 5-HT1A autoreceptors caused by increased synaptic 5-HT, thus preventing the negative feedback. This effect results in an increase in post-synaptic 5-HT neurotransmission and enhances the self-inhibitory effect of 5-HT reuptake. Moreover, it was revealed that the 5-HT1A agonists has a beneficial effect in improving cognitive functions in patients (Fakhoury, 2015; Yamada et al., 2023). Thus, SPARIs represent a class of novel antidepressants with remarkable advantages.

HEC113995PA·H_2_O is a highly-selective serotonin reuptake inhibitor and 5-HT1A receptor partial agonist. *In vitro* studies have revealed that it displays high affinity and antagonistic activity for the 5-HT transporter at the nanomolar level, and slightly weaker activity in agonizing 5-HT1A receptor than HEC61139 (Vilazodone). It exhibited comparable activity with Vortioxetine, which has been reportedly displaying a Ki value of 15.2 nM, EC50 value of 200 nM, and EMAX of 96% for the 5-HT1A receptor, as summarized in Table 2. HEC113995PA·H_2_O showed *in vitro* affinities or activities to other subtypes of 5-HT receptors, histamine receptors, and acetylcholine receptors at the micromolar level, but over 100-fold higher affinities towards SERT and the 5-HT1A receptor. As predicted, *in vitro* studies has obviously observed highly selective affinities and activities of HEC113995PA·H_2_O towards SERT and the 5-HT1A receptor. In vivo rodent studies, single, multiple, and chronic administrations of HEC113995PA·H_2_O significantly reduce the duration of immobility both in mice and rats in behavioral despair models, demonstrating promising antidepressant activity. It exhibited more potent efficacy than the drug targeting the same receptors, HEC61139 (Vilazodone), while also possessing an apparent sedative effect (unpublished data).

Therefore, we conducted this clinical trial to evaluate the safety, tolerability, and pharmacokinetic characteristics of HEC113995PA·H_2_O tablets in Chinese healthy subjects following single and multiple oral administrations. In addition, we assessed the impact of food intake on the pharmacokinetics and safety of HEC113995PA·H_2_O tablets.

## 2 Materials and methods

**FIGURE 2:**
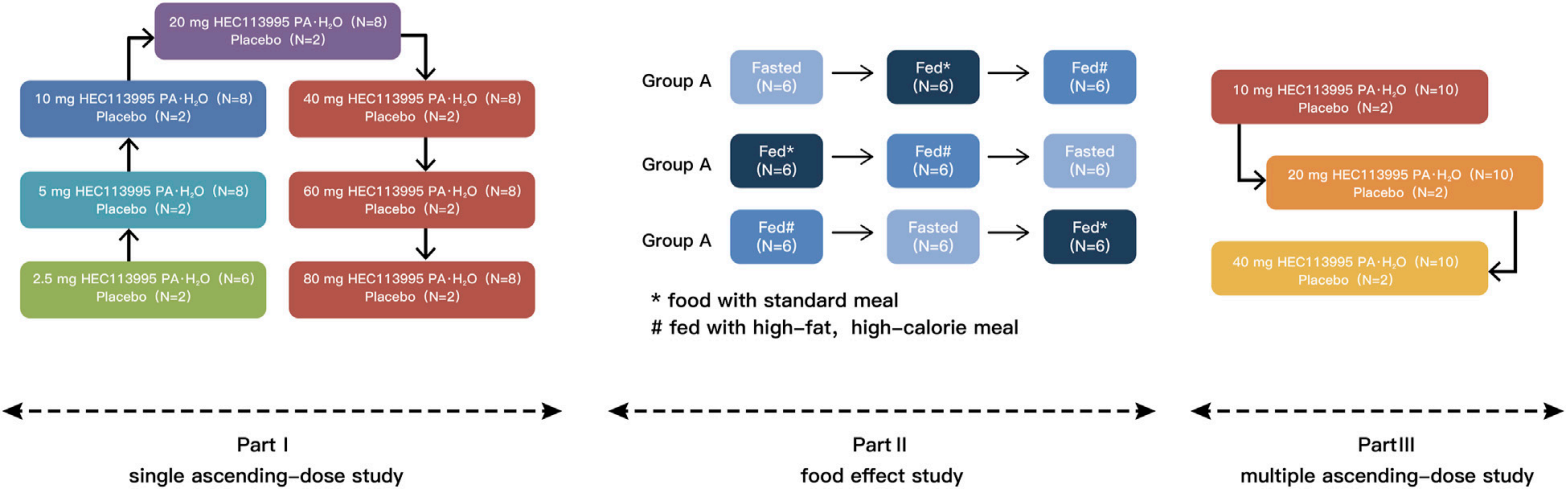
The scheme of study design.

This study scheme is composed of three sections: Sections 1–3 (Figure 2). Sections 2, 3 were randomized, double-blind, placebo-controlled, single and multiple ascending dose studies. Section 2 was a randomized, open-label, three-cycle, cross-over study to investigate the food effect of HEC113995PA·H_2_O. The study was conducted at the Phase I Clinical Research Center of Shanghai Xuhui Central Hospital, Shanghai, China, from July 2019 to August 2020. The study has been registered on http://www.chinadrugtrials.org.cn (Registration No.: CTR20190930 and CTR20200318 respectively). The study has been approved by the Ethics Committee of the Shanghai Xuhui Central Hospital and conducted in compliance with the Declaration of Helsinki and Good Clinical Practice. Formal written consent was obtained from every subject before enrolment in this study.

### 2.1 Participants

Participates who met the following inclusion criteria, and did not meet the exclusion criteria, were enrolled in the study.

#### 2.1.1 Inclusion criteria

1) Healthy males or females aged between 18 and 45 years, bodyweight ≥50 kg (males) or 45 kg (females), and body mass index (BMI) ranged between 18 and 28 kg/m^2^ (included);
2) Normal vital signs, physical parameters, 12-lead ECG and results of laboratory tests, and no apparent clinical symptoms;
3) Being able to utilize effective contraceptive measures throughout the study and for a period of 3 months following the administration of investigational products;

#### 2.1.2 Exclusion criteria

1) Individuals with serum creatinine, alanine aminotransferase, and aspartate aminotransferase levels ≥1.5 folds of the upper limit during the screening period;
2) Individuals testing positive for hepatitis B surface antigen, hepatitis C antibody, HIV antibody, or syphilis antibody at the time of screening;
3) Subjects diagnosed with clinically significant diseases;
4) Participants with a known history of allergy or allergic constitution to the test preparation, any of its components, or related preparations;
5) Any prescriptions or over-the-counter medication taken within 14 days prior to the study, or any medication taken within 28 days that inhibits or induces hepatic metabolism of the drug;
6) Individuals consuming foods or beverages containing caffeine, xanthine, alcohol, or grapefruit within 48 h before taking the study drug;
7) Drug or alcohol addicts, or heavy smokers;
8) Blood donation or blood loss >400 mL within 1 month before taking the study drug;
9) Individuals who plan to receive organ transplantation or have undergone organ transplantation;
10) Individuals who participated in other drug clinical trials within 3 months before randomization;
11) Subjects with other factors considered by the investigator to be ineligible for the trial.

### 2.2 Procedures

#### 2.2.1 Section I: single ascending-dose study

The study comprised a total of 7 dose groups (2.5, 5, 10, 20, 40, 60, and 80 mg groups, respectively). The first dose group (2.5 mg) consisted of 8 subjects, with 6 receiving HEC113995PA·H_2_O and 2 receiving placebo. Dose groups 2–7 each included 10 subjects, with 8 subjects receiving HEC113995PA·H_2_O and 2 subjects receiving placebo. Male and female participants were randomly assigned to the groups. Serial blood samples were collected before and after drug administration to determine the plasma concentration of pharmacokinetics (PK) and to evaluate the pharmacokinetic characteristics. The time points of blood collection and safety evaluation of PK in the second to seventh dose groups were adjusted based on the results of the first dose group. Drug safety and tolerability were evaluated on day 2 and 4 in the first dose group and on day 4 and 7 in the second to seventh dose groups. The trial was conducted in a dose-escalation manner, with progression to the next dose arm only after the safety of the previous arm had been evaluated. In the fourth group (20 mg group), in addition to blood samples, urine and stool samples were also collected for *in vivo* drug metabolic transformation studies.

#### 2.2.2 Section II: food effect study

A total of 18 healthy subjects of both genders, were assigned randomly to three sequences A, B, and C (n = 6). All subjects received HEC113995PA·H_2_O tablets, with a total of 3 times administered during the trial, once per cycle, and a washout period of ≥11 days. In sequence A, the order of administration was as follows: fasting, after a standard meal, and after a high-fat meal. In sequence B, the order of administration was after a standard meal, after a high-fat meal, and fasting. Sequence C subjects were administered in the following order: after a high-fat meal, fasting, and after a standard meal. During the fed periods, a single oral dose of HEC113995PA·H_2_O tablet 20 mg was administered within 30 min after consumption of a standard meal (total calories: approximately 600 kcal), or a high-fat, high-calorie meal (total calories: approximately 800 kcal–1,000 kcal, which derives about 150, 250, and 500–600 kcal from protein, carbohydrate, and fat, respectively). Serial blood samples were collected before and after each cycle for PK determination of plasma concentration and the evaluation of pharmacokinetic characteristics. Safety and tolerability were assessed on days 4 and 28.

#### 2.2.3 Section III: multiple ascending-dose study

Three drug doses (10, 20 and 40 mg) were utilized. A total of 36 healthy subjects of both genders were assigned randomly to the three groups (n = 12), with10 subjects taking HEC113995PA·H_2_O tablets and 2 taking placebo. The participants in the 10 mg dose group received a single dose daily in the morning after overnight fasting, or a single dose daily after a standard meal in the 20 mg and 40 mg dose groups for 10 consecutive days. Serial blood samples were collected within 0.5 h before drug administration on days 7, 8 and 9, and before and after drug administration on days 1 and 10 to determine plasma concentration and pharmacokinetic characteristics. Safety and tolerability were evaluated on days 4, 8, 13 and 15. The detailed trial groups and subject distribution were shown in Figure 1.

The trial was conducted in a dose-escalation manner, with the administration of the next dose arm only after the tolerability and safety of the previous arm had been fully evaluated. Following the assessments by both the investigator and the sponsor, the next dose level could be reverted to the previous dose group if deemed necessary. In such cases, twelve new healthy subjects (10 taking HEC113995PA·H_2_O tablets and 2 taking placebo) were enrolled to explore the maximum safe tolerated dose. Throughout the entire trial (including the SAD trial, FE trial and MAD trial), each subject was only allowed to participate in one dose group. Replacements were permitted only for participants who withdrew after randomization and did not receive any dose.

### 2.3 Safety assessment

Safety indicators were assessed through adverse events (AE), clinical laboratory tests (including blood routine, blood biochemistry, urine routine), vital signs monitoring (ear temperature), pulse, respiration and blood pressure in a sitting position), 12-lead electrocardiogram (ECG) and physical examinations. Adverse events (AE) and serious adverse events (SAE) were evaluated following the criteria outlined in National Cancer Institute Common Terminology Criteria for Adverse Events (NCI-CTCAE) version 5.0.

Any adverse events that occurred in all subjects during the clinical trial were recorded with the following details: AE name, occurrence time, end time, severity, whether it was classified as a SAE, severity criteria (if applicable), treatment measures and outcomes, actions taken on investigational drug due to the AE, and the causal relationship between the AE and administrated drug, etc. Each AE was recorded separately.

The subjects were under close monitoring at the clinical research center until the sample retention and safety evaluation were completed. During the safety trial, drug intolerance of a given dose was defined as follows: grade 2 or higher drug-related AEs occurred in more than half of the subjects, or grade 3 or higher drug-related AEs occurred in more than one quarter of the subjects. The dose escalation was halted after drug intolerance occurred.

### 2.4 Biological sample collection

In the first dose group of single ascending-dose study, PK blood samples were collected at 0.5 h before administration and 0.5, 1, 1.5, 2, 2.5, 3, 4, 5, 6, 8, 10, 12, 24, 36, 48, and 72 h after administration. In other SAD groups, MAD groups and food effect study, PK blood samples were collected at 0.5 h before administration, and 1, 2, 3, 4, 5, 6, 7, 8, 10, 12, 24, 48, 72, 96, 120 and 144 h after administration. Approximately 4 mL of venous blood was obtained from each collection and placed in a vacuum collection vessel containing K2EDTA anticoagulant. The blood was centrifuged and packed according to the instruction provided by the Biological Sample Detection and Analysis Unit. Briefly, all samples were centrifuged at 4°C, 1,500 g for 10 min within 0.5 h after blood collection. After centrifugation, the plasma was aliquoted into two cryogenic storage tubes. The first tube (Tube A) contained at least 0.8 mL of plasma for analytical testing, and the remaining plasma is allocated into the backup tube (Tube B). The plasma samples were stored at −80°C within 1 h after aliquoting for further analysis.

### 2.5 Bioanalytical procedures

The concentration of HEC113995PA·H_2_O in human plasma samples (K2EDTA) was quantitatively analyzed using the LC-MS/MS method. Plasma samples (50 μL) were processed using protein precipitation with 5 μL deuterated internal standard (HEC124659) and 100 μL acetonitrile. The Shimadzu LCMS-8060 system coupled with a GL Sciences Inc. InertSustain AQ-C18 HP column (50 × 2.1 mm, 3 μm) achieved chromatographic separation through gradient elution using 0.04% TFA aqueous solution (pH 2.1) and acetonitrile at 0.4 mL/min. ESI-positive mode detection employed optimized parameters: 300°C interface temperature, 250°C DL temperature, 3 L/min nebulizer gas, and 400°C heating block temperature.

The method showed linearity from 0.515 ng/mL (LLOQ, S/N > 10) to 1,030 ng/mL (ULOQ). Validation results revealed intra-assay precision ≤8.0% RSD with accuracy −3.9%–11.8%, and inter-assay precision ≤7.0% RSD with accuracy 0.3%–5.0%. All validation parameters including selectivity (signal contribution ≤20% of LLOQ), carryover (<20% LLOQ), dilution integrity (accuracy 85%–115%), matrix effects (CV ≤ 15%), and stability under various conditions met acceptance criteria. The maximum batch size was validated with maintained performance.

This fully validated method demonstrated appropriate sensitivity, precision, and robustness for clinical pharmacokinetic studies of HEC113995PA·H_2_O in human plasma samples.

### 2.6 Pharmacokinetic assessments

Using WinNonlin 7.0 for non-compartmental analysis (NCA), pharmacokinetic (PK) parameters were meticulously calculated based on the actual sampling times for plasma, urine, and fecal samples from each subject post-administration. These parameters encompassed a comprehensive range, including the area under the concentration–time curve (AUC), AUC from time zero (pre-dose) to the time of the last measurable concentration (AUC_0-t_), and AUC from time zero (pre-dose) to infinity (AUC_0-∞_). Additionally, maximum observed plasma concentration (Cmax), time to achieve maximum plasma concentration (Tmax), terminal elimination half-life (t_1/2_), elimination rate constant (Ke), apparent volume of distribution (Vd/F), clearance rate (CL/F), and mean residence time (MRT) were also determined. The AUC_0-t_ and AUC_0-∞_ values were computed using the linear trapezoidal rule method. Tmax and C_max_ were ascertained based on the actual observed data. In the context of the multiple ascending-dose study, both the degree of fluctuation (DF) and the accumulation ratio at steady state (Rac) were thoroughly analyzed.

### 2.7 Statistical analyses

Statistical analysis was meticulously conducted using SAS Software, version 9.4 (SAS Institute, Cary, NC, United States). The descriptive statistics were comprehensively represented, encompassing the arithmetic mean, standard deviation, coefficient of variation, median, maximum, minimum, and geometric mean of each dosage group. In the case of categorical variables, both frequency and percentage were methodically calculated to provide a clear summary. A threshold of P ≤ 0.05 was established as the criterion for statistical significance, ensuring rigorous evaluation of the data.

A mixed-effects model (Power Model) was employed to explore the dose proportionality of the pharmacokinetic response in healthy subjects following a single oral administration of HEC113995PA• H_2_O tablets, ranging from 2.5 to 80 mg. The Power Model is defined as: log(y) = β0 + β1*log (dose), in which 'y' represents the pharmacokinetic parameters (AUC_0-t_, Cmax, AUC_0-∞_). In this equation, β0 is the intercept, and β1 is the slope. For the statistical analysis using the Power model, log^10^ was treated as a fixed effect. The point estimate of β1 along with its 90% confidence intervals (CIs) were predicted. Dose proportionality was considered to be present within the studied dose range of HEC113995 PA·H_2_O if β1 is close to 1. The 90% CI is contained within the range [1 + ln (θ_L_)/ln(r), 1 + ln (θ_H_)/ln(r)], where θ_L_ and θ_H_ are typically 0.8 and 1.25, respectively, and “r” represents the dose ratio (80/2.5). A scatter plot of log (dose)-log(y) with the regression fit line was created to visualize these relationships.

In the food effect study, the pharmacokinetic (PK) parameters AUC_0-t_, AUC_0-∞_, and C_max_ under various dietary conditions were subjected to statistical analysis after logarithmic transformation. Subsequently, these results were reverted back using antilogarithmic transformation to determine the least-squares geometric mean, the point estimate of the mean ratio, and the 90% confidence interval for the ratio. For each PK parameter, the least-squares mean and the differences between the respective treatment groups (high-fat meal vs fasted, standard meal vs fasted, and high-fat meal vs standard meal) were meticulously calculated. The impact of food intake on the bioavailability of HEC 113995 was deemed negligible if the 90% confidence interval of the geometric mean ratio for AUC_0-t_, AUC_0-_∞, and C_max_ under fed conditions fell within the range of 80%–125% compared to those under fasted conditions.

## 3 Results

### 3.1 Demographic profile

A total of 121 eligible subjects were enrolled in the study, including 68 in Section 1, 18 in Section 2, and 35 in Section 3, respectively. Subject distribution is displayed in Figure 1. All subjects completed the study following the trial protocol. All subjects receiving drug administration were included in the safety analysis set and the full analysis set. The demographic profiles of all enrolled subjects is summarized in Table 1.

**TABLE 1:** Demographic profile of enrolled subjects.

	Single ascending-dose study								Food effect study			Multiple ascending-dose study			
	2.5 mg (N = 6)	5 mg (N = 8)	10 mg (N = 8)	20 mg (N = 8)	40 mg (N = 8)	60 mg (N = 8)	80 mg (N = 8)	Placebo (N = 14)	Group A	Group B	Group C	10 mg QD (N = 10)	20 mgQD (N = 9)	40 mgQD (N = 10)	Placebo (N = 6)
Age, years	25 (4.56)	25.5 (3.66)	29.1 (2.90)	29.5 (5.73)	26.5 (4.31)	28.5 (4.21)	26.5 (2.39)	27.3 (5.33)	26.5 (3.67)	29.8 (4.7)	30 (7.29)	27.4 (5.1)	24.2 (2.54)	31.5 (7.79)	30 (5.55)
Gender Male n (%) Female n (%)	4 (66.7%) 2 (33.3%)	7 (87.5%) 1 (12.5%)	8 (100%) 0	6 (75.0%) 2 (25.0%)	8 (100%) 0	7 (87.5%) 1 (12.5%)	8 (100%) 0	14 (100%) 0	6 (100%) 0	6 (100%) 0	6 (100%) 0	6 (60%) 4 (40%)	6 (66.7%) 3 (33.3%)	7 (70%) 3 (30%)	4 (66.7%) 2 (33.3%)
Height, cm	166.0 (4.6)	167.9 (3.4)	166.1 (2.9)	169.0 (5.9)	171.1 (5.8)	169.1 (5.0)	168.9 (4.6)	170.9 (6.9)	166.5 (4.7)	165.2 (6.3)	168.3 (9.5)	162.7 (6.3)	165.9 (8.4)	165.2 (8.2)	164.5 (6.9)
Weight, kg	61.9 (6.3)	65.9 (10.9)	56.7 (4.8)	65.61 (7.5)	67.95 (7.6)	63.96 (11.8)	69.99 (7.9)	65.99 (7.4)	62.87 (7.2)	60.63 (5.8)	66.72 (6.8)	64.08 (8.6)	61.09 (5.5)	61.07 (6.3)	61.35 (9.0)
BMI, kg/m^2^	22.45 (2.2)	23.35 (3.3)	20.6 (1.8)	23.03 (1.7)	23.18 (2.4)	22.21 (3.0)	24.58 (2.8)	22.59 (2.1)	22.93 (2.5)	22.42 (2.6)	23.62 (1.1)	23.98 (2.6)	22.21 (2.2)	22.38 (2.4)	22.52 (2.3)

Note: Data are expressed as mean (SD), except for gender, which is shown as n (%).

Abbreviation: BMI, body mass index.

#### 3.1.1 Pharmacokinetic properties

##### 3.1.1.1 Section I: single ascending-dose study

**FIGURE 3:**
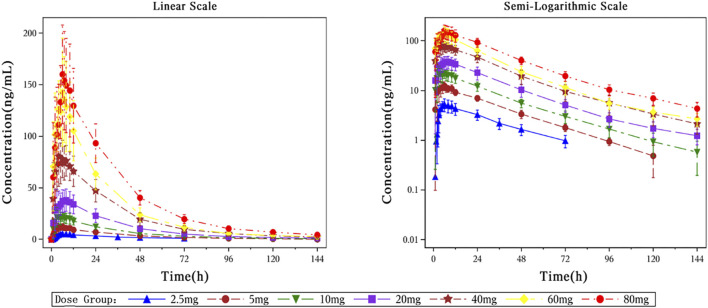
The Mean plasma concentration-time curves after a single dose of HEC113995PA·H_2_O.

The main PK parameters in each dose group after a single dose of HEC113995PA·H_2_O are summarized in Table 2, and the mean plasma drug concentration–time curves are shown in Figure 3.

**TABLE 2:** Main plasma PK Parameters in SAD.

SAD							
Pharmacokinetic paramaeters	2.5 mg (N = 6)	5 mg (N = 8)	10 mg (N = 8)	20 mg (N = 8)	40 mg (N = 8)	60 mg (N = 8)	80 mg (N = 8)
Plasma parameters							
C_max_ (ng/mL)	5.27 (1.52)	13.99 (3.86)	24.4 (7.71)	40.6 (12.36)	88.53 (23.08)	149.29 (49.29)	163.81 (46.23)
T_max_ (h)	6 (5,8)	5 (2,10)	6 (2,7)	8 (2,8)	5 (4,8)	5.5 (4,10)	6 (4,8)
t_1/2_ (h)	30.33 (4.52)	27.17 (2.11)	27.97 (5.26)	38.58 (8.39)	32.23 (4.19)	36.58 (17)	33.41 (8.53)
AUC_0-∞_ (h*ng/mL)	220.45 (53.22)	466.62 (68.05)	832.39 (199.68)	1,519.63 (440.93)	3,009.33 (750.39)	4,239.44 (1,015.52)	5,873.40 (1,080.89)
AUC_0-t_ (h*ng/mL)	176.92 (41.88)	438.67 (67.23)	801.23 (192.39)	1,447.92 (416.08)	2,910.33 (716.99)	4,095.39 (972.64)	5,652.39 (999.05)
Kel (1/h)	0.023 (0.003)	0.0256 (0.002)	0.0256 (0.005)	0.0187 (0.004)	0.0219 (0.003)	0.022 (0.008)	0.0219 (0.006)
Cl/F (L/h)	12.03 (3.52)	10.94 (1.75)	12.65 (3.13)	14.53 (5.78)	14.14 (4.06)	15.07 (4.58)	14 (2.41)
MRT(h)	44.81 (6.50)	39.78 (3.52)	39.13 (4.44)	42.21 (5.68)	38.14 (4.37)	35.27 (3.79)	40.48 (6.11)
Vz/F (L)	517.67 (123.58)	430.09 (85.24)	494.45 (74.07)	771.74 (206.53)	661.13 (226.34)	762.39 (317.08)	665.91 (156.29)

Notes: Data are expressed as mean (SD), except for T_max_, which is shown as median (min, max). **P < 0.01: The difference of the PK, parameter among different dose groups is considered to be statistically significant.

Abbreviations: C_max_, maximum observed plasma concentration; T_max_, time to maximum plasma concentration; t_1/2_, terminal elimination half-life; AUC_0_-_∞_, the area under the concentration-time curve from time zero to infinity; AUC_0-t_, the area under the concentration-time curve from time zero to the time of the last measurable concentration; V_z_/F, apparent distribution volume; Cl/F, clearance rate; MRT, mean residence time.

In this study, healthy subjects were orally administered a single dose of HEC113995PA·H_2_O at doses of 2.5, 5, 10, 20, 40, 60, and 80 mg. The median time to peak plasma concentration (Tmax) of HEC113995PA·H_2_O ranged between 5.00 and 8.00 h. The peak concentration (Cmax) and the systemic exposure of HEC113995PA·H_2_O increased along with higher doses. The geometric mean range for Cmax was 5.0996–157.3993 ng/mL. The geometric mean ranges for the systemic exposure (AUC_0–24h_, AUC_0-t_, AUC_0-∞_) were 88.3756–2,679.3141 hng/mL, 172.4890 to 5,577.1586 hng/mL, and 214.4500–5,790.9015 h*ng/mL, respectively. Parameters such as half-life (t_1/2_), elimination rate constant (Kel), mean residence time (MRT), apparent clearance (CL/F), and apparent volume of distribution (V_z_/F) did not show significant changes following increasing doses, with arithmetic mean values ranging from 27.17 to 38.58 h, 0.0187–0.0256 L/h, 35.27–44.81 h, 10.9380–15.0736 L/h, and 430.0893–771.7380 L, respectively. Overall, the pharmacokinetic characteristics in subjects after a single dose within the various dose groups appeared to be similar.

**FIGURE 4:**
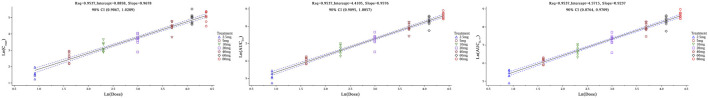
Dose linearity plots of C_max_ and AUC parameters in single ascending-dose study.

For Chinese subjects orally administered HEC113995PA·H_2_O at doses ranging between 2.5 and 80 mg, the correlation analysis among plasma pharmacokinetic parameters (Cmax, AUC_0-t_, and AUC_0-∞_) and dose showed that the slope estimates (β1) and their 90% confidence intervals (CI) for C_max_, AUC_0-t_, and AUC_0-∞_ were 0.9678 (90% CI: 0.9067–1.0289), 0.9576 (90% CI: 0.9095–1.0057), and 0.9237 (90% CI: 0.8764–0.9709), respectively, with a reference 90% CI evaluation standard of 0.9356–1.0644. The dose linearity plots are demonstrated in Figure 4. These data indicate that within the dose range of 2.5–80 mg, the exposure to HEC113995PA·H_2_O increases proportionally with the dose, implicating a dose-proportional relationship.

##### 3.1.1.2 Section II: food effect study

**FIGURE 5:**
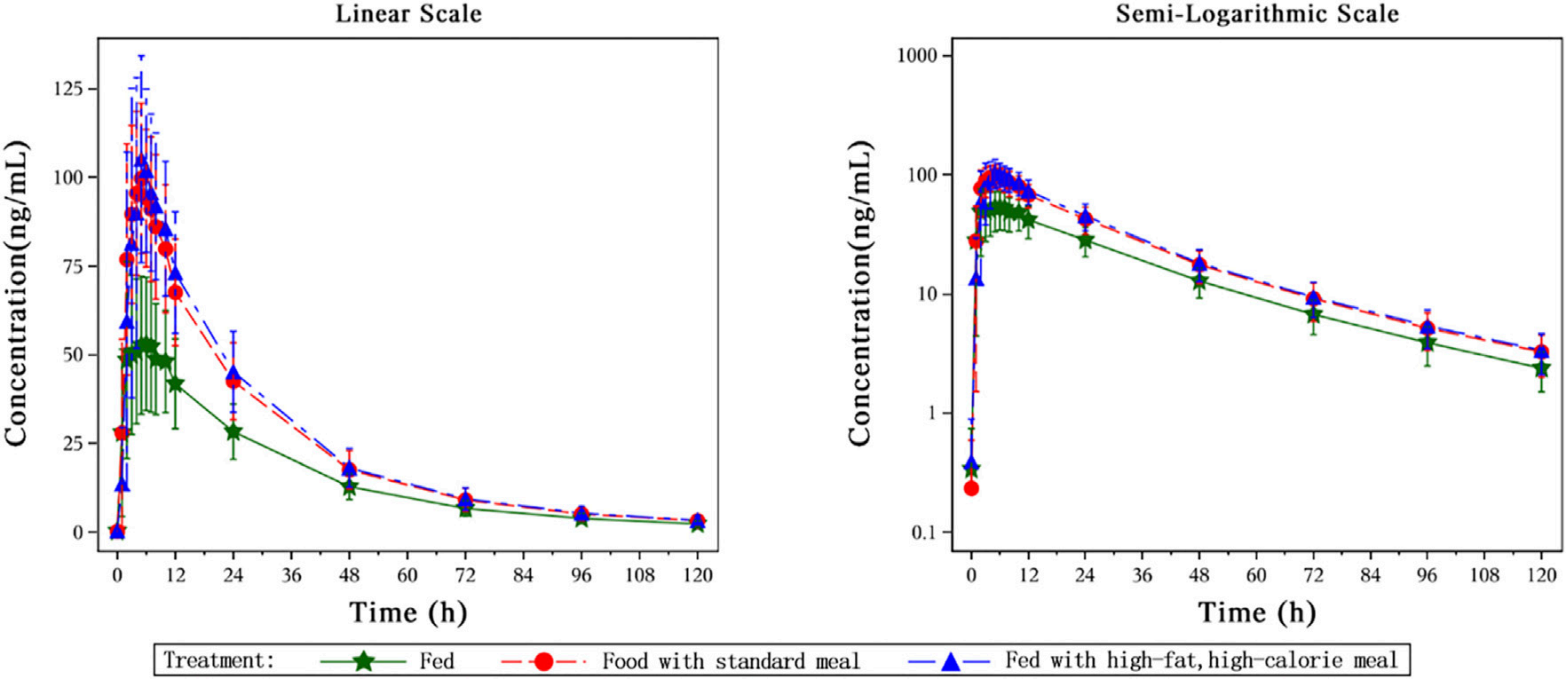
The mean plasma drug concentration–time curves under fasted and fed conditions.

The key PK parameters of HEC113995PA·H_2_O under fasted and fed conditions after a single oral dose of 20 mg HEC113995PA·H_2_O are listed in Table 3, and the mean plasma drug concentration–time curves under fasted and fed conditions are displayed in Figure 5.

**TABLE 3:** The main PK parameters of HEC113995PA·H_2_O under fasted and fed conditions after a single oral dose of 20 Mg HEC113995PA·H_2_O.

Pharmacokinetic paramaeters	Fasted (N = 18)	Fed with standard Meal (N = 18)	Fed with high-Fat,High-calorie Meal (N = 18)
T_max_ (h)	4 (2, 10)	5 (2, 7)	5 (2, 12)
C_max_ (ng/mL)	63.0 (22.92)	106.5 (17.9)	117.0 (23.8)
AUC_0-t_ (h*ng/mL)	1857.3 (474.8)	2, 834.6 (683.1)	2, 934.0 (644.8)
AUC_0-∞_ (h*ng/mL)	1959.5 (507.9)	2, 976.7 (733.6)	3, 085.3 (697.8)
CL/F (L/h)	10.8 (2.5)	7.1 (1.6)	6.8 (1.5)
Vz/F (L)	447.3 (113.2)	297.5 (75.9)	296.9 (68.5)
t_1/2(h)_	29.0 (4.5)	29.5 (5.2)	30.5 (4.1)
MRT (h)	38.3 (5.7)	35.4 (4.2)	35.8 (4.8)

Notes: Data are expressed as mean (SD), except for T_max_, which is shown as median (min, max). *P < 0.05: The difference of the PK, parameter under fasted and fedconditions is considered to be statistically significant.

Abbreviations: AUC_0-t,_ area under the concentration–time curve from time zero to the time of the last measurable concentration; AUC_0-∞_, area under the concentration–time curve from time zero to infinity; C_max_, maximum observed plasma concentration; T_max_, time to maximum plasma concentration; t_1/2_, terminal elimination half-life; V_Z_/F, apparent distribution volume; CL/F, clearance rate.

After single dose administration of 20 mg HEC113995PA·H_2_O tablet, food intake increased the systemic exposure AUC_0-∞_ and maximum concentration (C_max_) of HEC113995 PA·H_2_O by 52.15∼58.20% and 77.09∼93.45%, respectively, whereas no significant impacts on T_max_ (time to maximum concentration) and t_1/2_ (half-life) was found. Compared with drug administration after a standard meal, post high-fat-meal administration led to the increases of AUC_0-∞_ and C_max_ by 3.97% and 9.24%,respectively, but with no significant statistical difference (P > 0.05).

##### 3.1.1.3 Section III: multiple ascending-dose study

**FIGURE 6:**
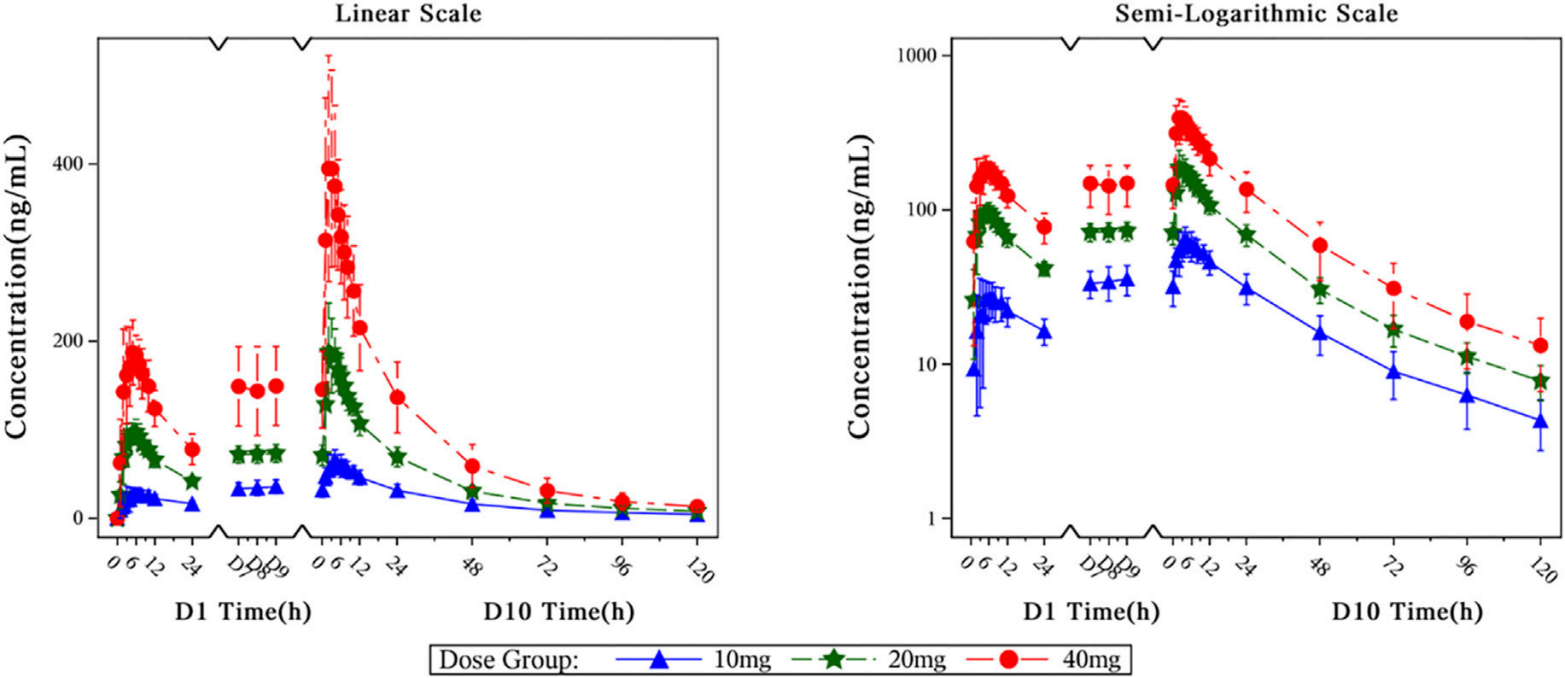
The mean plasma drug concentration–time curves at D1, D10 of 10–40 mg dose groups.

The main PK parameters of HEC113995PA·H_2_O in multiple-dose study at D1, D10 are presented in Table 4. The mean plasma drug concentration–time curves at D1, D10 of 10 mg, 20 mg and 40 mg dose groups are demonstrated in Figure 6.

**TABLE 4:** The main PK parameters of HEC113995PA·H_2_O in multiple ascending-dose study at D1, D10.

Pharmacokinetic paramaeters	10 mg (N = 10)	20 mg (N = 9)			40 mg (N = 10)	
	D1	D10	D1	D10	D1	D10
T_max_ (h)	5.5 (2,7)	4.5 (2,7)	5 (2,6)	2 (2,6)	5 (2,10)	2.5 (1,5)
MRT_0-∞_ (h)	39.96 (9.96)		25.29 (2.19)		24.30 (5.20)	
C_max_ (ng/mL)	30.6 (8.8)	—	104 (16.51)	—	208 (21.55)	—
AUC_0–24h_ (h*ng/mL)	485.78 (111.8)	—	1,529.64 (173.53)	—	2,929.48 (370.06)	—
AUC_0-t_ (h*ng/mL)	483.09 (111.45)	—	1,522.91 (172.9)	—	2,917.23 (367.15)	—
V_z_/F (mL)	350.36 (100.48)	—	188.9 (18.42)	—	191.7 (29.52)	—
Cl/F (mL/h)	9.40 (1.58)	—	8.1 (1.06)	—	8.8 (1.57)	—
t_1/2_ (h)		39.10 (6.94)		38.59 (8.93)		30.86 (6.79)
C_ss_min_ (ng/mL)	—	29.98 (7.7)	—	66.75 (9.34)	—	132.19 (40.3)
C_ss_max_ (ng/mL)	—	68.68 (12.6)	—	202.6 (39.27)	—	435.6 (87.5)
AUC_ss_ (h*ng/mL)	—	1,107.5 (178.6)	—	2,793.4 (365.2)	—	5,729.7 (1,252.0)
AUC_0-∞,ss_ (h*ng/mL)	—	2,485.8 (538.0)	—	5,471.5 (842.6)	—	10,542.9 (3,169.4)
CLss/F (L/h)		9.25 (1.56)		7.27 (0.97)		7.25 (1.4)
Vss/F (L)		521.3 (167.5)	—	402.1 (103.0)	—	320.8 (90.0)
Rac (AUC)	—	2.39 (0.64)	—	1.83 (0.16)	—	1.96 (0.35)
R_ac_Cmax_	—	2.41 (0.76)	—	1.96 (0.28)	—	2.1 (0.4)

Notes: Data are expressed as mean (SD), except for T_max_, which is shown as median (min, max). **P < 0.01: The difference of the PK, parameter among different dose groups is considered to be statistically significant.

Abbreviations: T_max_, time to maximum plasma concentration; t_1/2,_ terminal elimination half-life; MRT_0-∞,_ average retention time from zero to infinity; C_max_, maximum observed plasma concentration; AUC_0-∞,_ area under the concentration–time curve from time zero to infinity; AUC_0-t,_ area under the concentration–time curve from time zero to the time of the last measurable concentration; Vz/F, apparent distribution volume; Cl/F, apparent total body clearance after oral administration; C_ss_min,_ minimum observed plasma concentration (at steady state); C_ss_max,_ maximum observed plasma concentration (at steady state); AUC_0-∞_,_ss,_ area under the concentration–time curve from time zero to infinity (at steady state); AUC_0-t,ss,_ area under the concentration–time curve from time zero to the time of the last measurable concentration (at steady state); Vz/F_ss,_ apparent distribution volume (at steady state); CL/F_ss,_ apparent total body clearance after oral administration (at steady state); Rac accumulation ratio at steady state.

After healthy subjects took HEC113995PA·H_2_O tablets at various doses (10 mg, 20 mg, and 40 mg), plasma concentration of HEC113995PA·H_2_O increased along with drug dose. The trend of blood drug concentration over time was generally consistent between single and multiple administrations. Following a single administration in each dose group, the median time to peak concentration (T_max_) ranged between 5.00 and 5.50 h. The drug’s internal exposure increased with the dose. The relationships among the apparent volume of distribution (Vz/F), apparent clearance (CL/F), and dose were insignificant. After multiple administrations of various dose groups, the median range of steady-state peak time (Tss, max) in plasma varied between 2.00 and 4.50 h. The level of systemic exposure increased with the dose. After multiple administrations in each dose group, a certain degree of drug accumulation within the body was observed. The accumulation ratios (R_ac___Cmax_ and R_ac___AUC_) had arithmetic mean ranges of 1.9546–2.4058 and 1.8289 to 2.3919, respectively. After multiple administrations in each dose group, the results of the apparent volume of distribution (Vz/F), apparent clearance (CL/F), and half-life (t_1/2_) were close.

In the range from 10 to 40 mg, the PK in healthy subjects reached a steady-state after drug continuous administration for 7 consecutive days.

Healthy subjects receiving HEC113995PA·H_2_O tablets at doses between 20 and 40 mg following a standard meal, displayed no apparent gender difference in systemic exposure between single and multiple administrations. The gender difference analysis for the 10 mg dose group administered on an empty stomach showed no significant gender difference in systemic exposure; however, due to the low sample size, further confirmation is required.

The results of food effect trials indicated that post-meal administration exhibited better drug absorbance than fasting administration, with increased peak concentration and systemic exposure. Food intake had an impact on the pharmacokinetic characteristics of HEC113995PA·H_2_O.

Based on the results of gender difference analysis during the MAD phase, after receiving fasting administration of HEC113995PA·H_2_O tablets in the 10 mg dose group, subjects with different genders exhibited P-values greater than 0.05 in the T-test in terms of major pharmacokinetic parameters (except for the parameter AUCs, which had a P-value of 0.0495). However, because of the small sample size in this dose group (6 male subjects, 4 female subjects), the result of this analysis needs further confirmation. Healthy subjects in 20 mg and 40 mg dose groups did not show apparent gender differences in systemic exposure of the drug after post-meal administration of HEC113995PA·H_2_O.

### 3.2 Safety

#### 3.2.1 Section I: single ascending -dose study

A total of 65 adverse events were reported in the overall population of the safety analysis set, all of which were treatment-emergent adverse events (TEAE). The number (incidence) of subjects experiencing TEAEs were 36 (52.9%), including 1 case (16.7%) in 2.5 mg group, 3 cases (37.5%) in the 5 mg group, 0 case in the 10 mg group, 5 cases (62.5%) in the 20 mg group, 6 cases (75%) in the 40 mg group, 7 cases (87.5%) in the 60 mg group, 7 cases (87.5%) in the 80 mg group, and 7 cases (50%) in the placebo group. Among them, the number of subjects experiencing treatment-related adverse event (TRAE)was 33 cases (48.5%), including 13 cases (19.1%) of diarrhea, 8 cases (11.8%) of elevated triglycerides, 8 cases (11.8%) of dizziness, 5 cases (7.4%) of nausea, 5 cases (7.4%) of hyperuricemia, 2 cases (3.7%) of limb pain, 1 case (1.9%) of abdominal distension, 1 case (1.9%) of elevated conjugated bilirubin, 1 case (1.9%) of elevated serum bilirubin and 1 case (1.9%) of elevated serum creatine phosphokinase. Among them, the severity of elevated conjugated bilirubin was grade 2 according to CTCAE 5.0, while other TRAEs were grade 1 and spontaneously relieved without treatment. One subject (1.5%) received concomitant medication (metronidazole) for toothache as an adverse event, which was assessed as a significant AE.

#### 3.2.2 Section II: food effect study

During the period of FE trial, a total of 11 subjects (61.1%) experienced 20 TEAE incidents, in which 6 incidents occurred during fasting administration, 4 incidents (22.2%) during administration after a standard meal, and 5 (27.8%) incidents during administration after a high-fat meal. In total, 9 subjects (50.0%) experienced 13 TRAEs. Among them, 4 incidents (22.2%) occurred during fasting administration, 3 incidents (16.7%) during administration after a standard meal, 5 incidents (27.8%) during administration after a high-fat meal. The healthy subjects demonstrated good safety and tolerability of 20 mg HEC113995PA·H_2_O administrated when fasting, after a standard meal and after a high-fat meal.

The number (incidence) of subjects experiencing TRAE was included:Elevated blood uric acid in 4 cases (22.2%), distributed as follows: 1 case under fasting conditions, 2 cases after a standard meal, and 2 cases after a high-fat meal. Elevated blood triglycerides in 2 cases (11.1%), with 1 case under fasting conditions and 1 case after a high-fat meal. Nausea in 2 cases (11.1%), occurring as 2 cases under fasting conditions, 1 case after a standard meal, and 1 case after a high-fat meal. Elevated conjugated bilirubin in 1 case (5.6%) and elevated blood bilirubin in 1 case (5.6%), both occurring after a high-fat meal. All TRAEs were Grade 1 in severity and resolved spontaneously without treatment.

#### 3.2.3 Section III: multiple ascending-dose study

During the period of MAD trial, a total of 28 subjects (80.0%) experienced 95 TEAE incidents. In the HEC113995PA·H_2_O trial group, a total of 24 subjects (82.8%) experienced 85 TEAE incidents, including 8 subjects (80.0%), 8 subjects (88.9%) and 8 subjects (80.0%) in 10 mg, 20 mg and 40 mg trial groups, respectively. Among them, 19 subjects (65.5%) experienced 48 TRAEs, including 7 subjects (70.0%), 6 subjects (66.7%) and 6 subjects (60.0%) in 10 mg, 20 mg and 40 mg trial groups, respectively. In the placebo group, a total of 4 subjects (66.7%) experienced 10 TEAE incidents, among which, 3 subjects (50%) experienced 6 TRAEs.

In the MAD study, TEAEs related to the investigational product with ≥5% incidence were as follows:

• Dizziness (20.7%, 6/29): Distributed across dose groups as 1 case (10 mg), 1 case (20 mg), and 4 cases (40 mg).
• Nausea (17.2%, 5/29): 1 case (10 mg), 3 cases (20 mg), and 1 case (40 mg).
• Elevated blood triglycerides (17.2%, 5/29): 2 cases (10 mg) and 3 cases (40 mg).
• Headache (10.3%, 3/29): Exclusively observed in the 20 mg group.
• Dry mouth (10.3%, 3/29): All cases in the 10 mg group.
• Diarrhea (10.3%, 3/29): 2 cases (10 mg) and 1 case (20 mg).
• Terminal insomnia (10.3%, 3/29): Limited to the 20 mg group.
• Oral ulcer (6.9%, 2/29): 2 cases in the 10 mg group.
• Elevated gamma-glutamyl transferase (γ-GT, 6.9%, 2/29): 1 case each in 10 mg and 40 mg groups.
• Hyperuricemia (6.9%, 2/29): Both cases in the 40 mg group.

There are 9 subjects experienced Grade 2 AEs, of which 4 were deemed related to the investigational product. 3 cases (10.3%) in the HEC113995PA·H_2_O group: 1 case of elevated blood triglycerides in the 10 mg group, 1 case of elevated blood triglycerides and 1 case of elevated blood cholesterol in the 40 mg group. 1 case (16.7%) in the placebo group: allergic dermatitis. All other AEs in the MAD trial were Grade 1 in severity, with no Grade 3 or higher TEAEs related to the investigational product.

## 4 Discussion

The present Phase I clinical trial establishes fundamental pharmacokinetic and safety profiles for HEC113995PA·H_2_O, a novel dual-action agent targeting both serotonin reuptake inhibition (via SERT blockade) and 5-HT1A receptor partial agonism. This bifunctional pharmacodynamic profile positions HEC113995PA·H_2_O within an emerging class of antidepressants designed to synergistically modulate serotonergic neurotransmission through complementary mechanisms - potentially addressing limitations of conventional selective serotonin reuptake inhibitors (SSRIs) through enhanced receptor specificity and pathway modulation.

Our pharmacokinetic analysis reveals distinct advantages over Vilazodone, the prototypical SERT/5-HT1A dual-target agent. At equivalent 20 mg doses, HEC113995PA·H_2_O demonstrates 42% higher C_max_ (40.6 vs. 28.5 ng/mL) and threefold greater AUC_0-∞_ (1,519.6 vs. 502 h ng/mL), suggesting superior bioavailability and systemic exposure. The extended median t_1/2_ (27.17–38.58 h vs. Vilazodone’s 24.1 h) may confer clinical advantages through reduced dosing frequency requirements, a critical factor in antidepressant adherence. Notably, the delayed T_max_ (5–8 h) compared to Vilazodone’s 5-h peak suggests modified absorption kinetics that could influence both therapeutic onset and side effect profiles.

The pronounced food effect observed (52%–93% increases in exposure parameters) aligns with lipid-solubility characteristics common to psychotropic agents, likely mediated through enhanced lymphatic absorption or reduced first-pass metabolism. Importantly, the comparable exposure between standard and high-fat meals simplifies dietary recommendations, which has practical advantages over medications that requiring strict fat-content management. From a therapeutic optimization perspective, postprandial administration could strategically leverage this food effect to both enhance bioavailability and mitigate gastrointestinal AEs, mirroring strategies successfully employed with drugs like ziprasidone (Baumgartner et al., 2020; Choi et al., 2012; Khan, 2009; Vilazodone, 2020).

Dose proportionality across the 10–40 mg range and predictable accumulation ratios (Rac 1.83–2.41) indicate linear pharmacokinetics, supporting flexible dose titration in clinical populations. The achievement of steady-state by Day 7 compares favorably with conventional SSRIs (typically 5–7 days), suggesting this novel agent maintains equilibrium kinetics comparable to established therapies despite its dual mechanism. The stable Vz/F and CL/F across doses further reinforce its pharmacokinetic predictability.

Safety data reveal AE profiles consistent with serotonergic pharmacodynamics, where gastrointestinal (nausea, diarrhea) and neurological (dizziness, headache) symptoms mirror those of Vilazodone and other SERT-modulating agents. The transient grade 1–2 laboratory abnormalities (triglycerides, uric acid) warrant particular attention given their potential cardiovascular implications. While dietary confounders preclude definitive attribution, these findings echo observations with atypical antipsychotics where metabolic effects emerge with chronic use, suggesting the need for longitudinal monitoring in future trials.

The dual SERT/5-HT1A targeting strategy builds upon 3 decades of antidepressant development that has progressively moved from single-target (SSRIs) to multi-modal agents (e.g., vortioxetine’s 5-HT transporter/receptor polypharmacology). Our pharmacokinetic profile suggests HEC113995PA·H_2_O could potentially optimize the risk-benefit ratio through two mechanisms: 1) The partial 5-HT1A agonism may accelerate therapeutic onset by directly modulating autoreceptors, addressing the 4–6 week latency period characteristic of SSRIs; 2) Enhanced receptor specificity could reduce off-target effects associated with broader monoaminergic activity.

The proposed Phase II dosing strategy (10–40 mg postprandial) appropriately balances exposure optimization and tolerability concerns. However, the 40 mg dose’s 2.4-fold accumulation ratio suggests need for careful monitoring of chronic exposure, particularly given the molecule’s extended half-life. Future studies should incorporate therapeutic drug monitoring to identify potential metabolic polymorphisms affecting clearance.

These Phase I findings position HEC113995PA·H_2_O as a promising candidate in the evolving landscape of multimodal antidepressants. Its pharmacokinetic advantages - enhanced bioavailability, favorable food effects, and linear accumulation - coupled with a manageable safety profile, warrant investigation in patient populations. Subsequent trials should prioritize elucidating the relationship between 5-HT1A receptor occupancy and clinical response while monitoring long-term metabolic effects. The dual mechanism of action presents an opportunity to address critical unmet needs in depression treatment, particularly regarding treatment-resistant subtypes and functional recovery.

### 4.1 Limitations

One limitation of our study is that, as a phase I study, the number of subjects in each group was small and the maximum age was 45 years old, further studies are needed to fully evaluate the safety and tolerance of this drug. Furthermore, there were only 18 female subjects enrolled in the study, further studies need to enroll more female subjects to evaluate the effect in female.

## 5 Conclusion

HEC113995PA·H_2_O was well tolerated in healthy Chinese subjects in the single dose range of 2.5–80 mg and multiple doses range of 10–40 mg daily. These results support dose selection and further clinical evaluation of HEC113995PA·H_2_O for further clinical studies. Based on the safety and pharmacokinetic data, suggesting that dose levels of 10 mg, 20 mg, and 40 mg, with a recommendation for administering the drug after meals, may be considered during the phase 2 clinical trials.

## Data Availability

The original contributions presented in the study are included in the article/supplementary material, further inquiries can be directed to the corresponding author.
